# Acetylsalicylic acid reduces cigarette smoke withdrawal-induced anxiety in rats *via* modulating the expression of NFĸB, GLT-1, and xCT

**DOI:** 10.3389/fphar.2022.1047236

**Published:** 2023-01-09

**Authors:** Alaa M. Hammad, Lujain F. Alzaghari, Malek Alfaraj, Walid Al-Qerem, Wamidh H. Talib, Fawaz Alasmari, Haneen Amawi, F. Scott Hall

**Affiliations:** ^1^ Department of Pharmacy, College of Pharmacy, Al-Zaytoonah University of Jordan, Amman, Jordan; ^2^ Department of Clinical Pharmacy and Therapeutic, Applied Science Private University, Amman, Jordan; ^3^ Department of Pharmacology and Toxicology, College of Pharmacy, King Saud University, Riyadh, Saudi Arabia; ^4^ Department of Pharmacy Practice, Faculty of Pharmacy, Yarmouk University, Irbid, Jordan; ^5^ Department of Pharmacology and Experimental Therapeutics, College of Pharmacy and Pharmaceutical Sciences, University of Toledo, Toledo, OH, United States

**Keywords:** GLT-1, XCT, NFkB, anxiety-like behavior, acetylsalicylic acid

## Abstract

**Background:** Chronic exposure to cigarette smoke produces neuroinflammation and long-term changes in neurotransmitter systems, especially glutamatergic systems.

**Objective:** We examined the effects of cigarette smoke on astroglial glutamate transporters as well as NF-κB expression in mesocorticolimbic brain regions, prefrontal cortex (PFC) and nucleus accumbens (NAc). The behavioral consequences of cigarette smoke exposure were assessed using open field (OF) and light/dark (LD) tests to assess withdrawal-induced anxiety-like behavior.

**Methods:** Sprague-Dawley rats were randomly assigned to five experimental groups: a control group exposed only to standard room air, a cigarette smoke exposed group treated with saline vehicle, two cigarette smoke exposed groups treated with acetylsalicylic acid (ASA) (15 mg/kg and 30 mg/kg, respectively), and a group treated only with ASA (30 mg/kg). Cigarette smoke exposure was performed for 2 h/day, 5 days/week, for 31 days. Behavioral tests were conducted weekly, 24 h after cigarette smoke exposure, during acute withdrawal. At the end of week 4, rats were given either saline or ASA 45 min before cigarette exposure for 11 days.

**Results:** Cigarette smoke increased withdrawal-induced anxiety, and 30 mg/kg ASA attenuated this effect. Cigarette smoke exposure increased the relative mRNA and protein expression of nuclear factor ĸB (NFĸB) in PFC and NAc, and ASA treatment reversed this effect. Also, cigarette smoke decreased the relative mRNA and protein expression of glutamate transporter1 (GLT-1) and the cystine-glutamate transporter (xCT) in the PFC and the NAc, while ASA treatment normalized their expression.

**Conclusion:** Cigarette smoke caused neuroinflammation, alterations in glutamate transporter expression, and increased anxiety-like behavior, and these effects were attenuated by acetylsalicylic acid treatment.

## 1 Introduction

Cigarette smoking has a negative effect on health, resulting in massive health-related economic losses that include substantial morbidity and mortality (Prochaska and Benowitz, 2019). Despite the fact that cigarette smoking is a serious public health problem and well known by users to cause serious health problems, there are around one billion cigarette smokers globally. According to WHO, smoking leads to seven million deaths worldwide each year and the number is expected to increase in the upcoming years (Yang et al., 2019). Every year, over a third of smokers attempt to quit, but only about 3% succeed (Berry et al., 2013), making nicotine cessation treatments a serious unmet medical need.

Nicotine dependence can be defined as a pattern of psychological attributes that result in the compulsive and repetitive search for, and use of, nicotine (Association, 2014). Some attributes are certainly pre-existing, while others develop over the course of the addiction process. Nicotine dependence affects the reward system in the brain, producing calming, stress-reducing, or satisfied feelings (Kowiański et al., 2018), which are certainly quite different from the euphoria or “high” typical of some other drugs of abuse. Non-etheless, ample data link nicotine dependence to the effect of nicotine on the brain reward system through increasing dopamine secretion in neurons originating in the ventral tegmental area (VTA) that project to the nucleus accumbens (NAc) and many other brain regions (Schilström et al., 1997; Wonnacott et al., 2005), in a manner similar to other drugs of abuse. In addition, nicotine induces glutamate release in the VTA, the NAc, prefrontal cortex (PFC), and amygdala (Morel et al., 2019), in a manner that is also similar to other drugs of abuse. Adaptations in glutamatergic systems occur in response to repeated nicotine exposure that involve astrocytes. It has been reported that adolescent female rats are more likely to self-administer nicotine than adolescent male rats and nicotine intake is higher in females (Lynch, 2009), although not all studies find sex-dependent effects (Chen et al., 2007; Schassburger et al., 2016). Most studies do find sex-dependent differences in nicotine responses showing that females are more sensitive. Importantly, another previous study suggested that nicotine had a greater rewarding effect in adolescent female rats than in adolescent male rats (Xue et al., 2020). Nicotine also produces more pronounced behavioral sensitization in female than male rats (Harrod et al., 2004). Consequently, we opted to use female rats in our study.

Astrocytes are glial cells that regulate the cellular absorption and release of glutamate, e.g., glutamate homeostasis, thereby affecting the development of drug dependence (Miguel-Hidalgo, 2009). Presynaptic glutamate release raises glutamate concentrations in the synaptic space to more than 1 mM, and because glutamate is not metabolized extracellularly, it is absorbed by transporters found in astrocytes, including glutamate transporter 1 (GLT-1) and the cysteine/glutamate antiporter (xCT), that act to limit the duration of synaptic action and diffusion of extracellular glutamate (Veruki et al., 2006). Previous studies have shown that changes in glutamate homeostasis in the PFC and NAc contribute to the reinstatement of drug-seeking behavior (Kalivas, 2009). GLT-1 is the most well-known of the five glutamate transporters, accounting for 90% of glutamate removal from synapses, which protects neurons from excitotoxicity and apoptosis (Kanner, 1993). Because of this role of GLT-1 in glutamate homeostasis, it is strongly associated with glutamate contributions to addiction (Roberts-Wolfe and W. Kalivas, 2015; Suárez-Pozos et al., 2020). Chronic nicotine exposure down-regulates GLT-1 and causes an increase in extracellular glutamate concentrations in synapses, ultimately contributing to increased cigarette smoking (Linker et al., 2019). Numerous studies have shown that GLT-1 upregulation can have therapeutic effects that contribute to smoking cessation (Roberts-Wolfe and W. Kalivas, 2015; Bechard and Knackstedt, 2019).

xCT is an antiporter, reliant on L-cysteine, that also contributes to glutamate homeostasis. This transporter mediates the exchange between extracellular L-cysteine, and intracellular L-glutamate, thereby increasing extracellular glutamate levels. Previous studies showed that the expression of xCT gene and protein is decreased by chronic nicotine exposure (Alasmari et al., 2021; Hammad et al., 2021), which may be a homeostatic mechanism that compensates for exaggerated glutamate release in response to acute nicotine exposure. Consequently, xCT expression is important for regulating baseline glutamate levels in the brain (Rao et al., 2015; Marczylo, 2020). Numerous studies have shown that xCT upregulation can also be a good target for smoking cessation (D’souza and Markou, 2011; Rao and Sari, 2014). Chronic nicotine consumption causes oxidative stress, which inhibits astrocyte glutamate transporters (Berríos-Cárcamo et al., 2020). Recent research suggests that cue-induced restoration of nicotine self-administration and chronic nicotine exposure in rats are linked with higher TNF-α levels in the PFC and NAc (Namba, 2019; Quintanilla et al., 2021).

Many of the negative consequences of smoking involve immune-inflammatory system impacts (Rom et al., 2013). Cigarette smoke exposure activates the molecular cascade that results in the production and release of NF-κB (Goncalves et al., 2011). The NF-κB pathway is one of the primary pathways behind smoking-induced activation of inflammatory cells. Cigarette smoke activates NF-κB *in vitro*, in cell culture, and *in vivo*, in animal models of cigarette smoke exposure (Gebel and Müller, 2001; Zhong et al., 2006). NF-κB affects cell survival and has a role in the development of mood disorders (Wohleb et al., 2011; Wohleb et al., 2013; Ramirez et al., 2016). Changes in mood and affect are also characteristic of acute responses to nicotine as well as nicotine withdrawal.

Acetylsalicylic acid (aspirin; ASA) is a non-steroidal anti-inflammatory medication frequently used to treat pain. ASA works to reduce the synthesis of prostaglandin E2 and pro-inflammatory cytokines by inhibiting the cyclooxygenase COX-1 and COX-2 enzymes (Israel et al., 2021). Importantly, ASA upregulates GLT-1 protein levels after chronic ethanol intake and chronic oral nicotine intake, blocks relapse to binge drinking, and inhibits nicotine reinstatement in female rats (Quintanilla et al., 2020; Israel et al., 2021). ASA also increases GLT-1 levels in the PFC (Quintanilla et al., 2021) and may also suppress the NF-κB pathway (Dąbek et al., 2010). To explore the relationships between neuroimmune states, glutamatergic systems, and neuroplasticity, this study investigated the effect of cigarette smoke exposure and treatment with ASA on NF-κB, GLT-1, and xCT relative mRNA and protein levels in mesocorticolimbic brain regions, i.e., the PFC and NAc. Moreover, the effect of cigarette exposure as well as ASA treatment on anxiety-like behavior was also examined.

## 2 Materials and methods

### 2.1 Animals

Forty female adult Sprague-Dawley rats weighing 180–250 g were bred at Al-Zaytoonah University of Jordan (ZUJ). Animals were grouped and housed in a vivarium room, kept on a 12-h/12-h light/dark cycle, and maintained under controlled temperature (23°C ± 2°C) and humidity (50% ± 5%) conditions. The animal protocol for this work was approved by the Animal Care and Use Committee of ZUJ, and all work was conducted in accordance with the Helsinki guidelines for animal research (Ashcroft, 2008). Animals had free access to food and water throughout the experiment. At the beginning of the experiment, rats were housed one animal per cage. The rats were divided randomly into five groups: Control Group (*n* = 8), Nicotine Group (**NIC**) (*n* = 8), Nicotine and ASA 15 mg/kg Group (**NIC/ASA15**) (*n* = 8), Nicotine and ASA 30 mg/kg Group (**NIC/ASA30**) (*n* = 8), and ASA 30 mg/kg Group (**ASA30**) (*n* = 8).

### 2.2 Drugs

LD Blue cigarettes (Liggett Ducat, .6 mg of nicotine, .8 mg of tar, and .01 mg of carbon monoxide) were purchased from a local marketplace. ASA (extra pure, BP, USP) was purchased from Hangzhou Soy MedTech Co., Ltd. ASA was dissolved in water, adjusted with Tris-base to pH 7.2 to protect against gastric damage.

### 2.3 Cigarette smoke exposure

**FIGURE 1:**
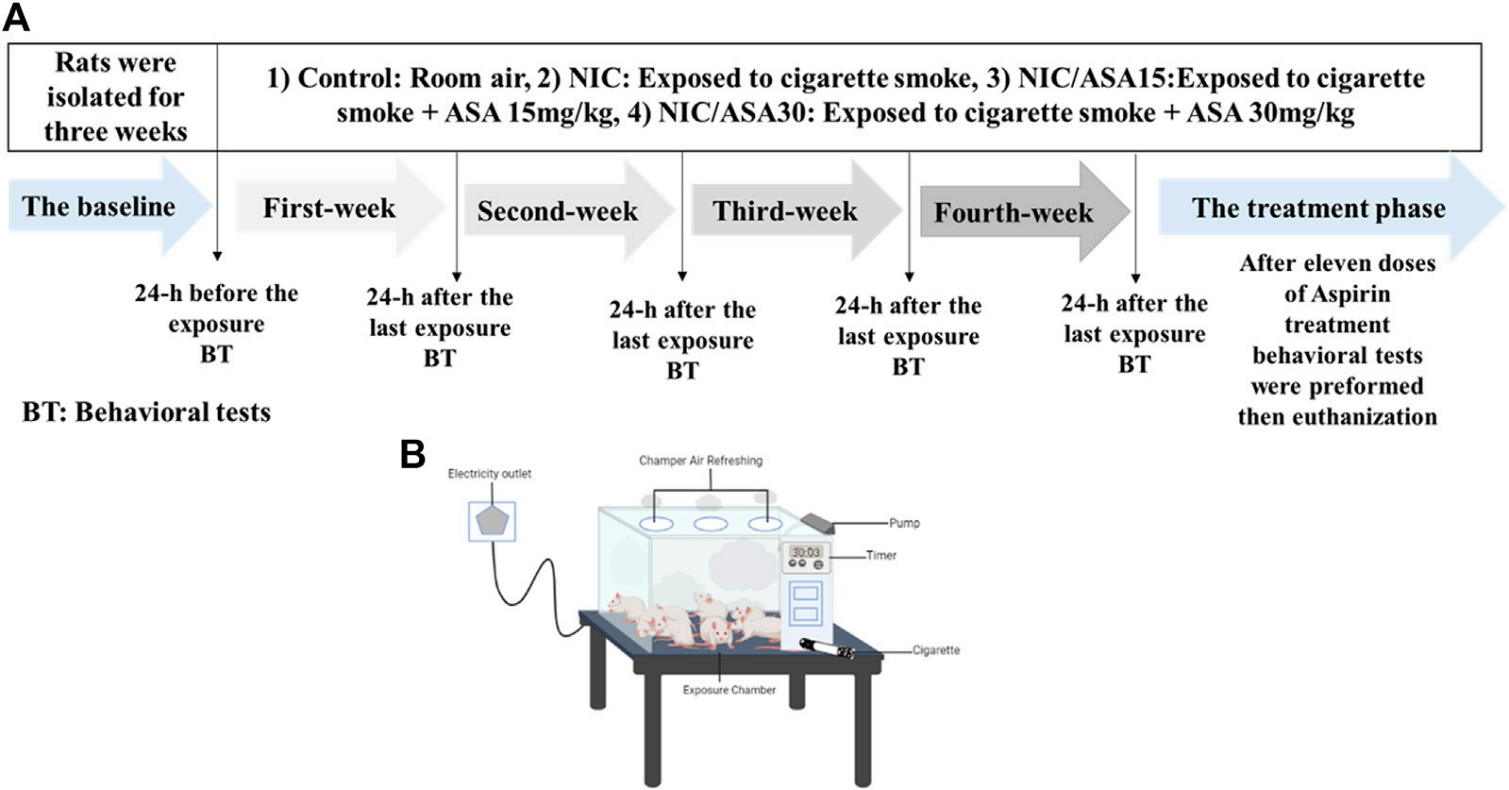
**(A)** Experimental timeline for cigarette exposure, ASA treatment and behavioral testing; **(B)** Diagram illustrating the apparatus used for cigarette exposure smoke.

The timeline for cigarette smoke exposure, ASA treatment, and behavioral tests is illustrated in Figure 1A. Although the cigarette smoke exposure method was passive, whole-body exposure, the level of exposure more closely approximates the levels produced by active smoking, and the effects are presumed to be primarily mediated by inhalation. All nicotine groups were exposed to cigarettes 2 h/day, 5 days/week, for 4 weeks, after which time rats received a daily oral gavage (OG) 45 min before cigarette exposure for 11 executive days, as follows: NIC groups received tris-base; NIC/ASA15 received a daily oral gavage of 15 mg/kg of ASA dissolved in tris-base; NIC/ASA30 received a daily oral gavage of 30 mg/kg ASA dissolved in tris-base. The control and ASA30 groups were exposed to room air during the exposure process and received a daily oral gavage of tris-base and ASA (30 mg/kg), respectively, for eleven executive days. Cigarette smoke exposure was accomplished by placing the rats inside an exposure chamber illustrated in Figure 1B. The exposure chamber is acrylic (40 cm × 40 cm × 40 cm) with a door in the upper portion so the rats can be placed inside the chamber, and then the chamber sealed during the smoke exposure. Twelve rats were placed in the apparatus together during each exposure. The door has three openings in the top for air circulation with an additional inlet where the cigarette is placed. The exposure chamber has a pump connected to it, which has been modified and programmed to allow smoke to enter the chamber from the cigarette *via* the smoke tube. A timer is connected to the pump controller to control the puff duration, and inter-puff intervals. In this experiment, a 3 s puff with a 30 snd inter-puff interval was specified with repeated cycles for the duration of the exposure (Shao et al., 2019). At the beginning of the exposure, two cigarettes were burned at once in order to saturate the exposure chamber after the rats were placed inside, after which the regular cycles of cigarette smoke exposure began. Twelve cigarettes were used within the 2-h exposure session. Carbon monoxide (CO) levels in the exposure chamber were monitored using an electrochemical sensor (Monoxor Ⅱ, Bacharach Inc. PA). The level of CO was kept around 700 ppm throughout the exposure process. Accordingly, if CO levels dropped under 500 ppm more cigarettes were used, and if CO levels rose above 899 ppm the pump was stopped for 1–2 min to bring CO levels back to the required range. Cotinine and nicotine levels were measured in our previous experiment using identical methods, the plasma concentration of nicotine was 222.98 ng/ml, and the plasma cotinine concentration was 376.12 ng/ml (Alhusban et al., 2022).

### 2.4 Behavioral tests

Behavioral OF and LD tests were conducted 24 h after the end of each week of cigarette exposure. During the baseline (before exposure) rats had free access to the apparatus (OF) for 10 min for 3 days to habituate them to the testing apparatus. There was no habituation for the LD test. On day three of habituation, the session was digitally recorded to provide a baseline for each rat’s performance. At the beginning of the behavioral tests, the OF field test was done first followed by the LD test. The test order was reversed for the second time point and reversed again for each subsequent test for the rest of the experiment. All of the tests were carried out during the daytime. As for ASA (30 mg/kg) behavioral tests were only conducted at the beginning of the experiment to measure the baseline and following an administration of 11 daily doses of ASA 30 mg/kg.

The OF apparatus is a white polyvinyl chloride plastic board square arena (50 cm × 40 cm × 25 cm) with a center zone in the middle of the box marked using permanent marker (20 × 25 cm). Each test session lasted for 20 min. Between each subject, the open-field area was cleaned with water. A video camera was placed above the apparatus. Each behavioral test was recorded and an observer blinded to the experimental conditions measured overall distance traveled and time spent in the center zone from observation of the digital recordings (Kraeuter et al., 2019).

The LD apparatus consists of two connected compartments (40 cm × 40 cm × 30 cm), one of which is illuminated (light/aversive area) and the other of which is dark (dark/safe area), with an opening in the wall separating them (7.5 cm × 5 cm) (Kulesskaya and Voikar, 2014). A table lamp light was used to illuminate the bright compartment (233 lx). A camera was mounted above the LD apparatus using a tripod. Rats were placed in the light compartment away from the door and then allowed to freely explore both compartments (Acevedo et al., 2014). Rats were placed in the apparatus for 10 min. Initial placement was on the light side. The time spent in the light compartment and latency to enter the dark chamber were measured. After each subject, both chambers were cleaned with water.

### 2.5 Brain tissue harvesting

After the final behavioral test, rats were euthanized using diethyl ether, decapitated with a guillotine and their brains were immediately removed and stored at −80°C until processing. Using the Rat Brain Atlas, brain regions of interest (the PFC and the NAc) were identified (Paxinos and Watson, 2006) and dissected on a cryostat (JUNG CM 1500, Leica) set at −20°C (right side samples were analyzed for mRNA and left side for protein).

### 2.6 Quantitative real-time polymerase chain reaction (qRT-PCR)

Total RNA was extracted from PFC and NAc samples using Direct-zol™ RNA MiniPrep Kit (ZYMO-RESEARCH, United States; cat# R2052), according to the manufacturer’s protocol. Four µg of total RNA from each sample was used for cDNA synthesis. Reverse transcription (RT) was performed using PrimeScript™ RT Master Mix from TAKARA, according to manufacturer’s protocol. The concentration and purity of RNA and cDNA samples were recorded for each sample using Quawell NanoDrop (Sunnyvale, CA, United States). The cDNA was diluted to 100 ng/L. One µl of diluted cDNA was used for each sample for qRT-PCR analysis, according to the manufacturer’s protocol. Table 1 describes the primer sequences used in the experiment. A threshold cycle number (CT) for each sample was obtained from a Prime Pro 48 Real-Time PCR System (Techne, United Kingdom) and used to evaluate the relative amount of target mRNA in experimental groups with those of control subjects using the 2^−ΔΔCT^ method (Livak and Schmittgen, 2001; Hamadneh et al., 2021).

**TABLE 1:** Primer sequence used for qPCR (Hammad et al. 2021).

Gene	Primer	Sequence
glt-1	F	5’-GAGCATTGGTGCAGCCAGTATT-3’
	R	5'-GTTCTCATTCTATCCAGCAGCCAG-3’
xct	F	5’-ACCTTTTGCAAGCTCACAGCAA-3’
	R	5’-AGCAGGAGAGGGCAACAAAGAT-3’
Nf-κb	F	5’-TGCCGAGTAAACCGGAACTC-3’
	R	5’-CAGCCAGGTCCCGTGAAATA-3’
β-actin	F	5’-ATCTGGCACCACACCTTC-3’
	R	5’-AGCCAGGTCCAGACGCA-3’

### 2.7 Western blot

Brain samples from the PFC and NAc were lysed using 1% SDS lysis buffer. Equal quantities of isolated proteins were mixed with 5X laemmli loading dye and separated on 10% polyacrylamide gels (SDS-PAGE). Proteins were then transferred to polyvinylidene fluoride membranes (PVDF, cat# sc-3723) using a Trans-Blot Turbo apparatus (Trans-Blot Turbo™ Transfer System, BIO-RAD). Membranes were blocked with 3% nonfat dried milk in TBST (50 mM Tris-HCl; 150 mM NaCl, pH 7.4; .1% Tween 20) for 30 min at 4°C. Membranes were incubated overnight at 4°C with one of the following primary antibodies: Rabbit Anti-EAAT2 (GLT-1) antibody (1:5000, ab205247; Abcam), Rabbit Anti-xCT antibody (1:5000, ab175186, Abcam), or Rabbit Anti-NF-κB p65 antibody (1:5000, ab76311, Abcam). Mouse Anti-β-Tubulin antibody was used as a loading control (1:5000, ab6046, Abcam). After incubation overnight, the primary antibody was removed, the membrane then was washed five time for 5 min each time in TBST, followed by addition of the secondary antibody (Goat Anti-Rabbit IgG H&L (HRP) for 120 min. After 2 h, the secondary antibody was removed and the membrane was incubated with SuperSignal West Pico Chemiluminescent substrate for imaging using a ChemiDoc™ Imaging System (BIO-RAD). An Image Lab 6.0.1 system was used to quantify the observed bands, and the levels were expressed as a percentage of the measured protein/β-tubulin ratio relative to the control groups (100% control-value).

### 2.8 Statistical analysis

Data were expressed as means and standard errors of the means (SEM). Two-way repeated measures ANOVA, followed by One-way ANOVA and Bonferroni multiple comparisons, were used to analyze data for behavioral tests followed by Tukey’s multiple comparisons. One-way ANOVA was used to analyze relative mRNA expression and relative protein expression. All statistical analyses were based on a *p* < .05 level of significance, using GraphPad Prism version 9.0.

## 3 Results

### 3.1 Effect of cigarette smoke exposure and ASA treatment on OF behavior

**FIGURE 2:**
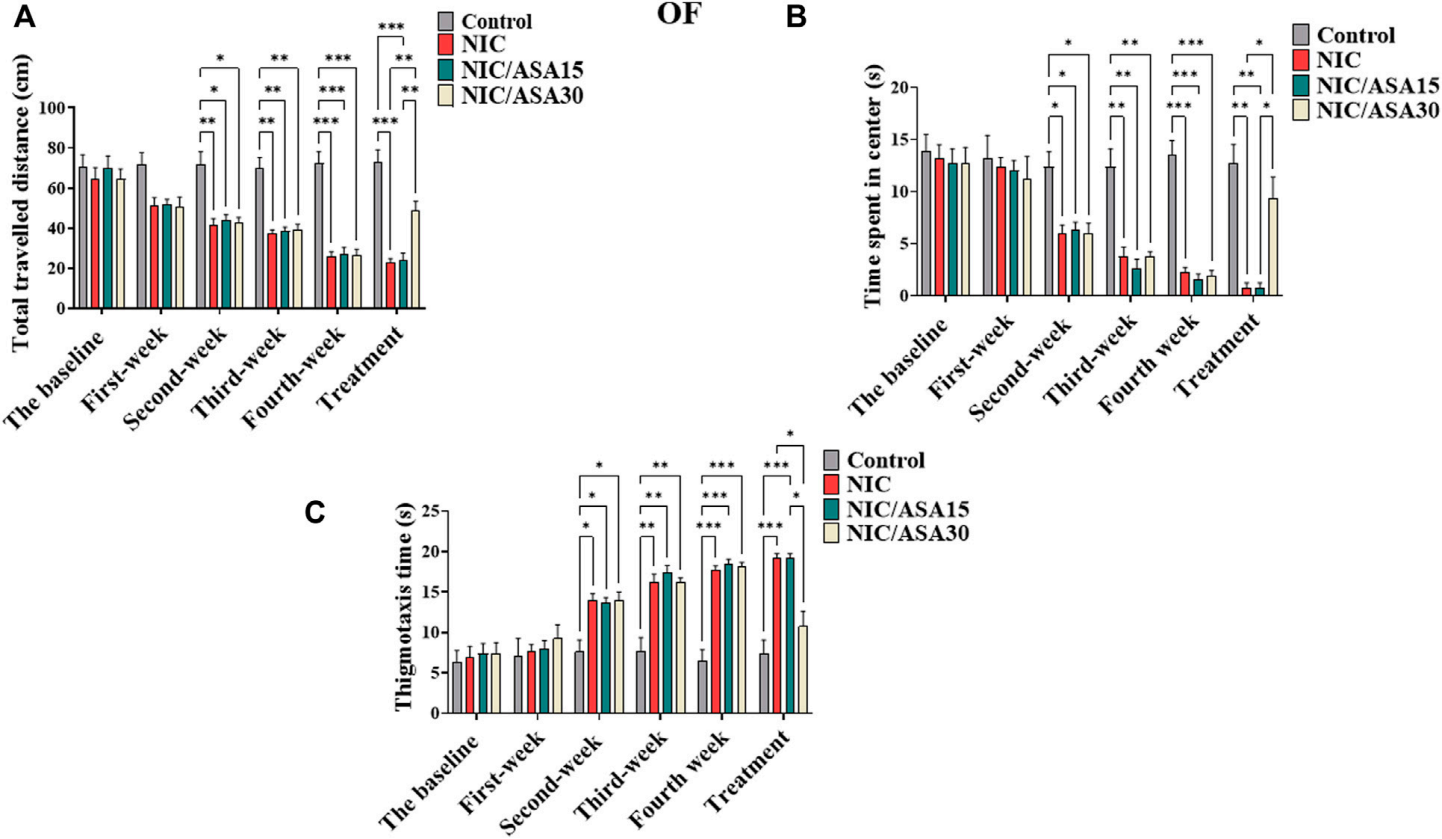
Behavioral testing in the OF test (data are presented as mean ± SEM). **(A)** Distance traveled in the OF in Control, NIC, NIC/ASA15, and NIC/ASA30 rats; **(B)** Time spent in the center of the OF in Control, NIC, NIC/ASA15, and NIC/ASA30 rats; **(C)** Thigmotaxis (time in the periphery of the open field) in Control, NIC, NIC/ASA15, and NIC/ASA30 rats (*p < .05, **p < .01, ***p < .001; *n* = 6 for each group).

Cigarette smoke exposure induced withdrawal-like anxiety in the OF, which appears over the weeks of exposure in terms of total distance travelled as shown in Figure 2A. Total distance travelled decreased over the 4 weeks of exposure to cigarette smoke in the NIC, NIC/ASA15, and NIC/ASA30 groups compared to the Control group. This difference was partially reversed by treatment with 30 mg/kg ASA, but not 15 mg/kg ASA, in week five of testing. The statistical significance of these effects was shown by repeated measures two-way ANOVA that revealed a significant main effect of Week [F (Berry et al., 2013; Berríos-Cárcamo et al., 2020) = 52.82, *p* < .0001], a significant main effect of Treatment [F (Berry et al., 2013; Berríos-Cárcamo et al., 2020) = 18.64, *p* < .0001], and a significant Week × Treatment interaction [F (15, 140) = 9.59, *p* < .0001]. Bonferroni multiple comparisons revealed significant decreases in total distance travelled at weeks 2, 3, and 4 in the NIC, NIC/ASA15, and NIC/ASA30 groups compared to the Control group. This effect was reversed by treatment with 30 mg/kg ASA, but not 15 mg/kg ASA, such that there a significant increase in total distance travelled in the NIC/ASA30 group compared with the NIC and NIC/ASA15 groups for week 5 (Figure 2A). A similar pattern was observed for time spent in center of the OF. Repeated measures two-way ANOVA revealed a significant main effect of Week [F (Berry et al., 2013; Berríos-Cárcamo et al., 2020) = 73.80, *p* < .0001], a significant effect of Treatment [F (Berry et al., 2013; Berríos-Cárcamo et al., 2020) = 9.992, *p* = .0001], and a significant Week × Treatment interaction [F (15,140) = 11.26, *p* < .0001]. Bonferroni multiple comparisons confirmed that there were significant decreases in the time spent in center zone of the OF at weeks 2, 3, and 4 in the NIC, NIC/ASA15, and NIC/ASA30 groups compared with the Control group. This effect was reversed by treatment with 30 mg/kg ASA, but not 15 mg/kg ASA. The results show a significant difference in time spent in center zone of the OF in the NIC and NIC/ASA15 groups compared with the NIC/ASA30 (Figure 2B). In the same manner, thigmotaxis (time in the periphery of the open field) was increased over the 4 weeks of exposure to cigarette smoke in the NIC, NIC/ASA15, and NIC/ASA30 groups compared to the Control group (the period during which there was no ASA treatment). Repeated measures two-way ANOVA revealed a significant main effect of Week [F (Berry et al., 2013; Berríos-Cárcamo et al., 2020) = 12.98, *p* < .0001], a significant effect of Treatment [F (Berry et al., 2013; Berríos-Cárcamo et al., 2020) = 10.73, *p* = .0001], and a significant Week × Treatment interaction [F (15,140) = 81.42, *p* < .0001]. Bonferroni multiple comparisons confirmed that there were significant increases in thigmotaxis time of the OF at weeks 2, 3, and 4 in the NIC, NIC/ASA15, and NIC/ASA30 groups compared with the Control group. This effect was reversed by treatment with 30 mg/kg ASA, but not 15 mg/kg ASA. The results show a significant difference in thigmotaxis time of the OF in the NIC and NIC/ASA15 groups compared with the NIC/ASA30 after ASA treatment (Figure 2C).

### 3.2 Effect of 30 mg/kg ASA on OF behavior

Treatment with 30 mg/kg ASA alone had no effect on the total distance travelled in the OF compared with the Control group. This was confirmed by repeated measures two-way ANOVA, which revealed no significant effect of Week [F (Kalivas, 2009; Prochaska and Benowitz, 2019) = .8839; ns] or Treatment [F (Kalivas, 2009; Prochaska and Benowitz, 2019) = .01505; ns], and no significant Week × Treatment interaction [F (Kalivas, 2009; Prochaska and Benowitz, 2019) = .2049; ns] (results not shown). There was also no effect of ASA alone on the time spent in the center zone. Treatment with 30 mg/kg ASA had no effect on the time spent in the center zone of the OF compared with the Control group. This was confirmed by repeated measures two-way ANOVA, which revealed no significant effect of Week [F (Kalivas, 2009; Prochaska and Benowitz, 2019) = .7857; ns], or Treatment [F (Kalivas, 2009; Prochaska and Benowitz, 2019) = .1248; ns], and no significant Week × Treatment interaction [F (Kalivas, 2009; Prochaska and Benowitz, 2019) = .1623; ns] (Supplementary Figures S1A, B, respectively).

### 3.3 Effect of cigarette smoke exposure and ASA treatment on behavior in the LD task

**FIGURE 3:**
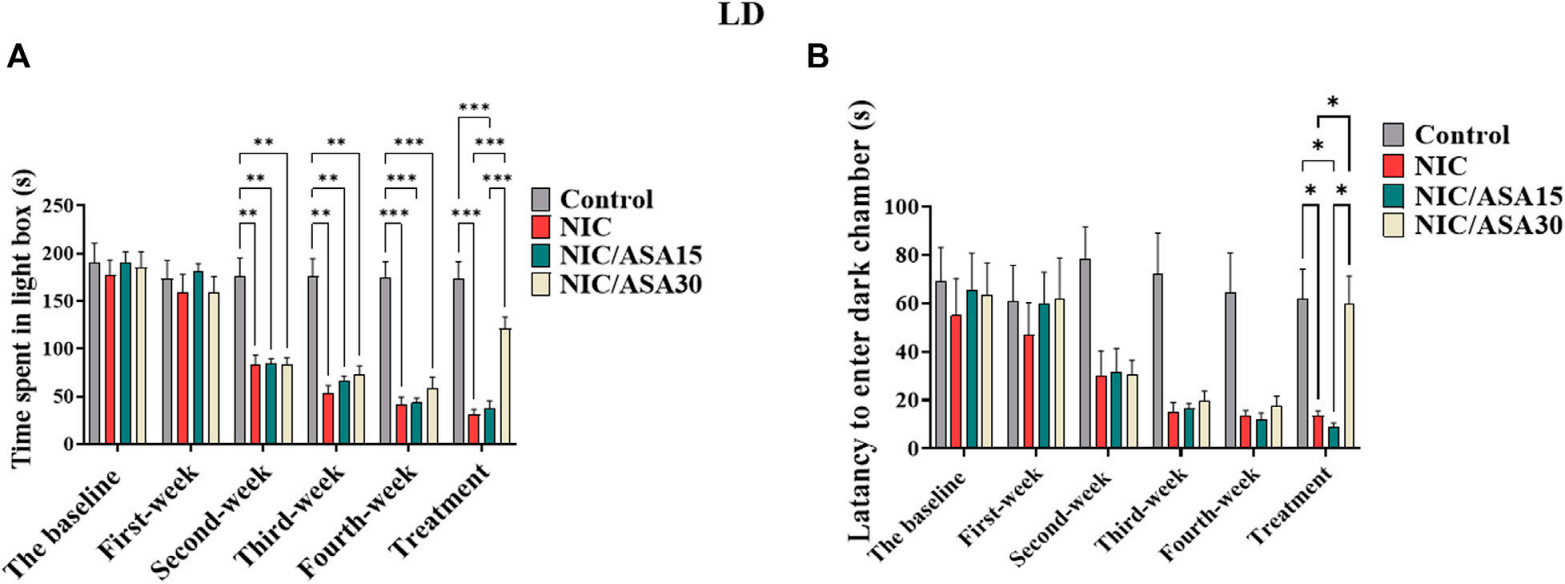
Behavioral testing in the LD test (data are presented as mean ± SEM). **(A)** Time spent in light chamber of the LD box in control, NIC, NIC/ASA15, and NIC/ASA30 rats; **(B)** Latency to enter the dark chamber of the LD box in Control, NIC, NIC/ASA15, and NIC/ASA30 rats (*p < .05, **p < .01, ***p < .001; *n* = 6 for each group).

Cigarette smoke exposure caused withdrawal-induced anxiety in the LD task that developed progressively over the first 4 weeks of exposure, seen as reductions in the time spent in the light compartment as shown in Figure 3A. This effect was reversed by treatment with 30 mg/kg ASA. This pattern of effects was confirmed by repeated measures two-way ANOVA, which revealed a significant main effect of Week [F (3,28 = 82.33, *p* < .0001], a significant main effect of Treatment [F (Berry et al., 2013; Berríos-Cárcamo et al., 2020) = 9.541, *p* = .0002], and a significant Week × Treatment interaction [F (15, 120) = 11.14, *p* < .0001]. Bonferroni multiple comparison tests revealed a significant decrease in time spent in light chamber at weeks 2, 3, and 4 in the NIC, NIC/ASA15, and NIC/ASA30 groups compared to the Control group. This effect was reversed by 11 days of treatment with 30 mg/kg ASA. There was a significant difference in time spent in light chamber in the NIC, and NIC/ASA15 groups compared with the NIC/ASA30 and Control groups in Week 5 (Figure 3A). A similar pattern was observed for latency to enter dark chamber, because of reduced movement and freezing when the animals were placed in the lit portion of the apparatus. Repeated measures two-way ANOVA revealed a significant main effect of Week [F (Berry et al., 2013; Berríos-Cárcamo et al., 2020) = 9.51, *p* < .0001], a significant effect of Treatment [F (Berry et al., 2013; Berríos-Cárcamo et al., 2020) = 6.15, *p* = .0039], and a significant Week × Treatment interaction [F (15,140) = 2.24, *p* = .0094]. Bonferroni multiple comparisons confirmed that there was a significant decrease in the latency to enter dark chamber in NIC and NIC/ASA15 compared to Control and ASA 30 mg/kg groups after 11 days of ASA 30 mg/kg treatment (Figure 3B).

### 3.4 Effect of 30 mg/kg ASA on behavior in the LD task

Treatment with 30 mg/kg ASA did not affect the time spent in the light chamber compared with the Control group. This was confirmed by repeated measures two-way ANOVA, which revealed no significant effect of Week [F (Miguel-Hidalgo, 2009; Prochaska and Benowitz, 2019) = .2422; ns] or Treatment [F (Miguel-Hidalgo, 2009; Prochaska and Benowitz, 2019) = .02797; ns], and no significant Week × Treatment interaction [F (Miguel-Hidalgo, 2009; Prochaska and Benowitz, 2019) = .9940; ns] (results not shown). There was also no effect on the frequency of stretching. This was confirmed by repeated measures two-way ANOVA, which revealed no significant effect of Week [F (Miguel-Hidalgo, 2009; Prochaska and Benowitz, 2019) = .1692; ns], or Treatment [F (Miguel-Hidalgo, 2009; Prochaska and Benowitz, 2019) = .2459; ns], and no significant Week × Treatment interaction [F (Miguel-Hidalgo, 2009; Prochaska and Benowitz, 2019) = .3807; ns] (Supplementary Figures S1C, D, respectively).

### 3.5 Effect cigarette smoke exposure and ASA treatment on glt-1, xct, and nf-κb mRNA expression in the PFC

**FIGURE 4:**
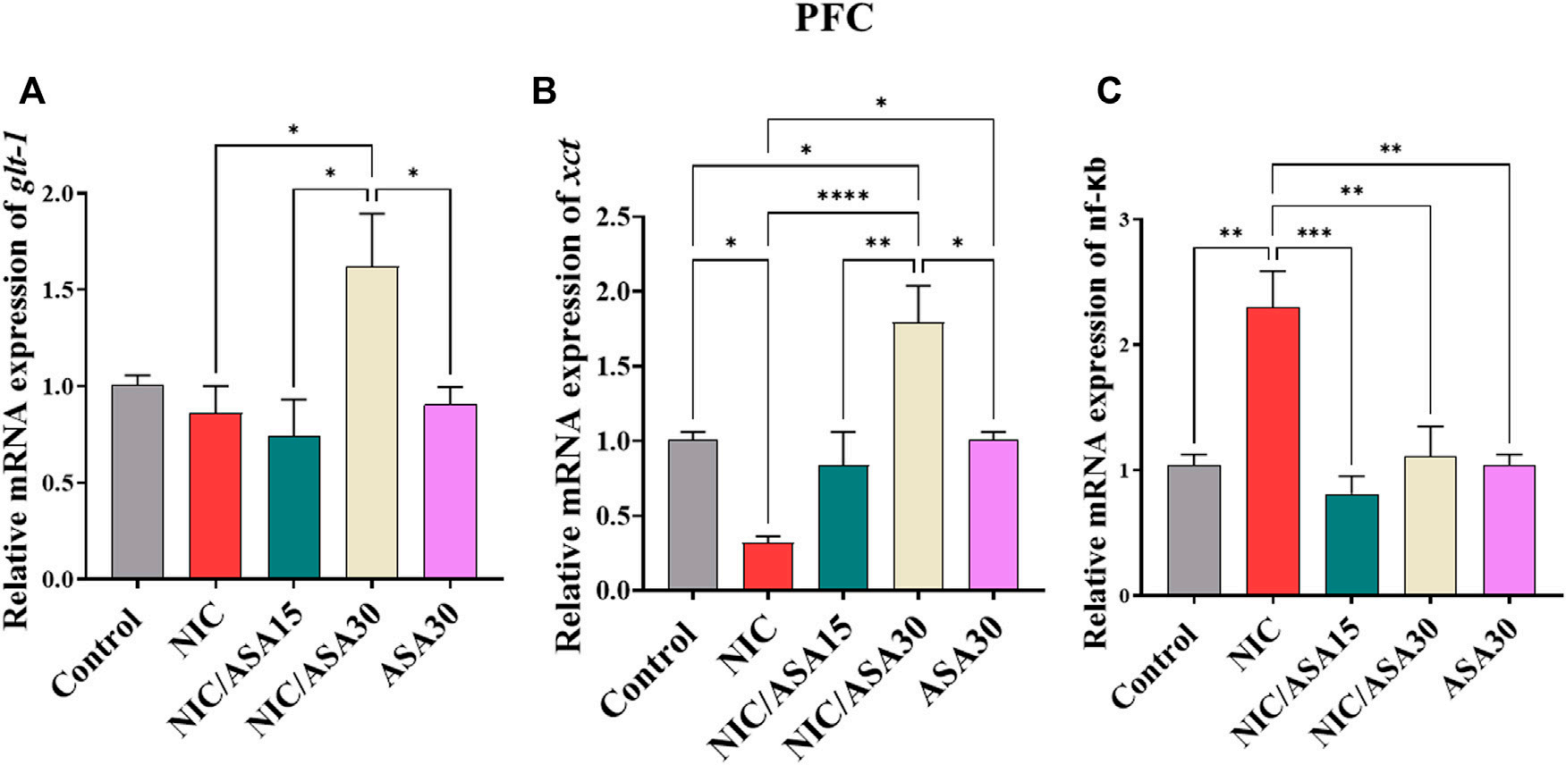
Relative glutamate transporters mRNA expression in PFC brain regions after treatments (data expressed as mean ± SEM). **(A)** glt-1; **(B)** xct; **(C)** nfκB (*p < .05, **p < .01, ***p < .001; *n* = 5 for each group).

There were no significant effects of cigarette smoke exposure for 31 days on relative *glt-1* mRNA expression in the PFC. However, relative *glt-1* mRNA expression was upregulated in NIC/ASA30 group compared to the NIC, NIC/ASA15 and ASA30 groups. This effect was confirmed by one-way ANOVA followed by Tukey’s multiple comparison (Figure 4A; [F (Association, 2014; Linker et al., 2019) = 4.195, .0126]). Interestingly, cigarette smoke exposure decreased relative *xct* mRNA expression in the PFC and this decrease was reversed by treatment with 30 mg/kg ASA, but not 15 mg/kg ASA, as shown in Figure 4B. Similar to relative *glt-1* mRNA expression, *xct* mRNA expression was upregulated in NIC/ASA30 group compared to the Control, NIC, NIC/ASA15 and ASA30 groups. This pattern of effects was confirmed by one-way ANOVA followed by Tukey’s multiple comparison [F (Association, 2014; Linker et al., 2019) = 11.81, *p* < .0001; Figure 4B]. Cigarette smoke exposure increased relative *nf-κB* mRNA expression in the PFC, and this increase was reversed by treatment with 15 mg/kg or 30 mg/kg ASA as shown in Figure 4C. This pattern was confirmed by one-way ANOVA followed by Tukey’s multiple comparison that showed an increase in relative *nf-κB* mRNA expression in the NIC group compared to the Control, NIC, NIC/ASA15, NIC/ASA30 and ASA30 groups [F (Association, 2014; Linker et al., 2019) = 9.962, *p* = .0001; Figure 4C].

### 3.6 Effect of cigarette smoke exposure and ASA treatment on glt-1, xct, and nf-κb mRNA expression in the NAc

**FIGURE 5:**
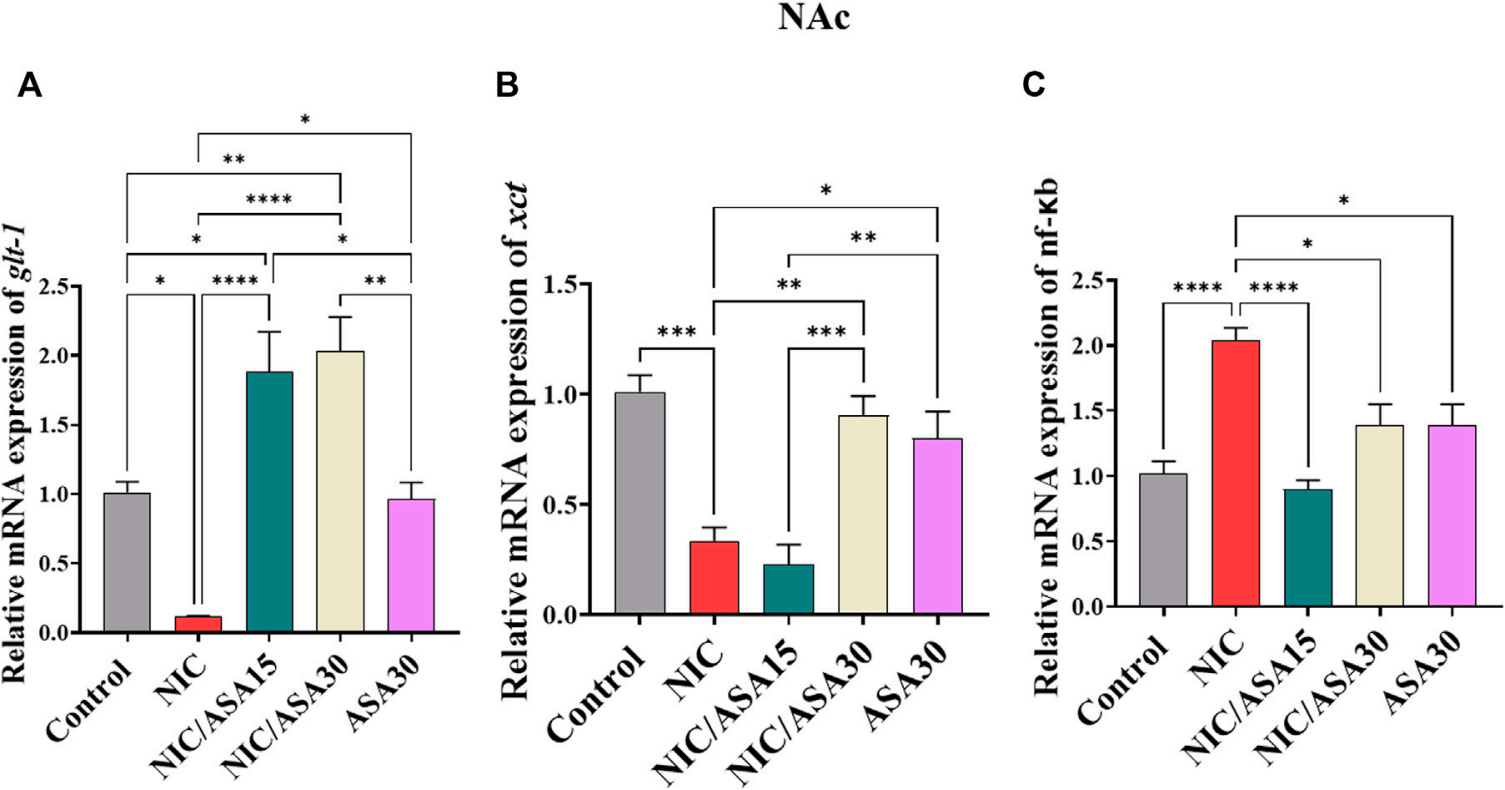
Relative glutamate transporters mRNA expression in NAc brain regions after treatments (data expressed as mean ± SEM). **(A)** glt-1; **(B)** xct; **(C)** nfκB (*p < .05, **p < .01, ***p < .001; *n* = 5 for each group).

Cigarette smoke exposure for 31 days decreased relative *glt-1* mRNA expression in the NAc and this decrease was reversed by 15 mg/kg and 30 mg/kg ASA treatment (Figure 5A). This pattern of effects was confirmed by one-way ANOVA followed by Tukey’s multiple comparison [F (Association, 2014; Linker et al., 2019) = 18.38, *p* < .0001; Figure 5A]. Relative *glt-1* mRNA expression was downregulated in the NIC group compared to the Control, NIC/ASA15, NIC/ASA30 and ASA30 groups. Moreover, relative *glt-1* mRNA expression was upregulated in the NIC/ASA15 and NIC/ASA30 groups compared to the Control and ASA30 groups.

A similar pattern of effects was found for *xct* mRNA expression in the NAc, where cigarette smoke exposure for 31 days decreased relative *xct* mRNA expression, although this decrease was reversed by 30 mg/kg ASA, but not 15 mg/kg ASA (Figure 5B). This pattern of effects was confirmed by one-way ANOVA followed by Tukey’s multiple comparison [F (Association, 2014; Linker et al., 2019) = 15.57, *p* < .0001; Figure 5B]. Relative *xct* mRNA expression was downregulated in the NIC group compared to the Control, NIC/ASA30 and ASA30 groups.

Finally, cigarette smoke exposure for 31 days increased relative *nf-κB* mRNA expression in the NAc and this effect was reversed by treatment with 15 mg/kg and 30 mg/kg ASA (Figure 5C). This pattern of effects was confirmed by one-way ANOVA followed by Tukey’s multiple comparison [F (Association, 2014; Linker et al., 2019) = 12.93, *p* < .0001; Figure 5C]. Relative *nf-κB* mRNA expression was upregulated in the NIC group compared to the Control, NIC/ASA15, NIC/ASA30, and ASA30 groups.

### 3.7 Effect of cigarette smoke exposure and ASA treatment on GLT-1, xCT, and NF-kβ protein expression in the PFC

**FIGURE 6:**
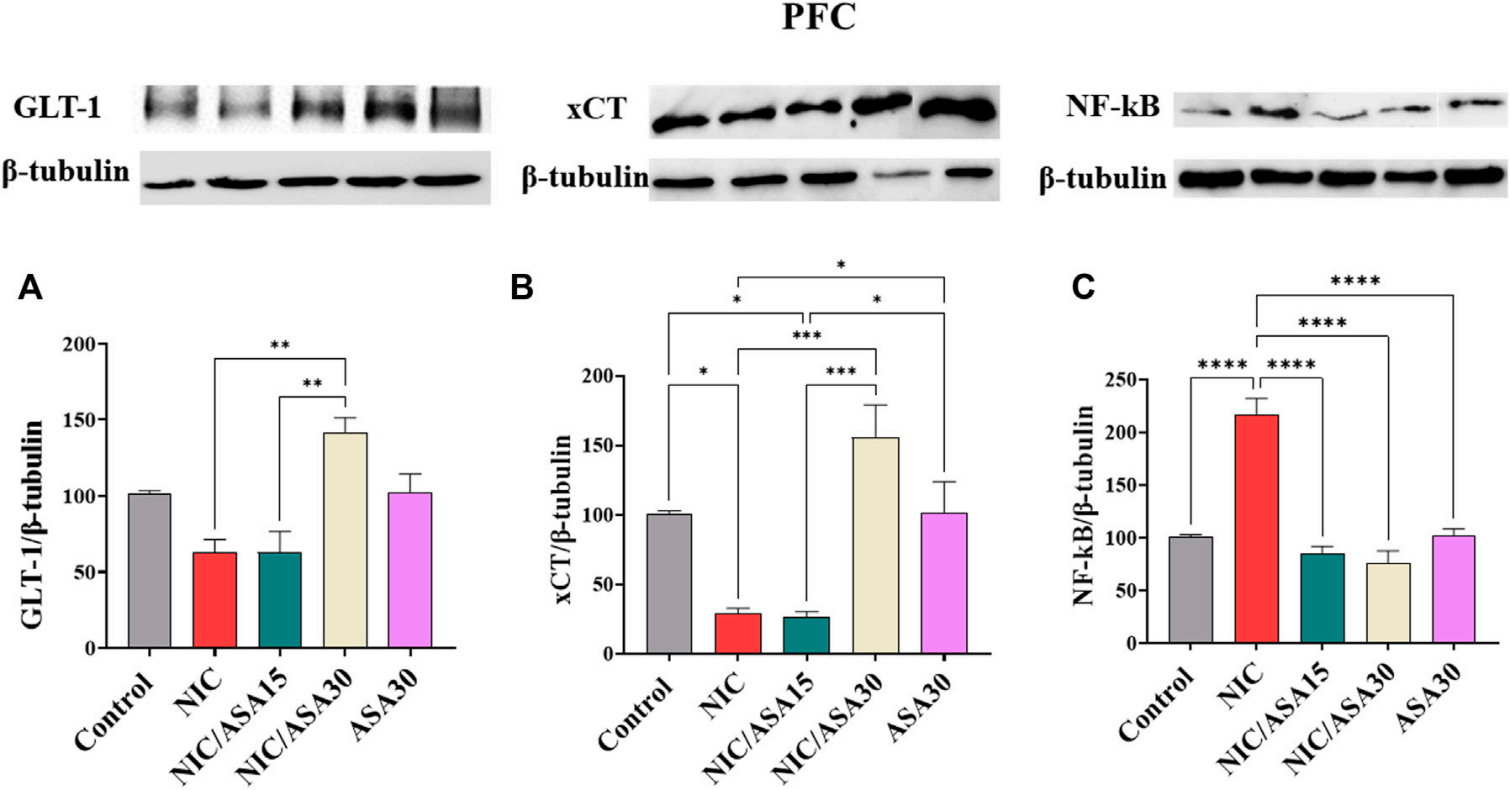
Glutamate transporters level in PFC brain regions after treatments (data expressed as mean ± SEM). **(A)** GLT-1; **(B)** xCT; **(C)** NFκB (*p < .05, **p < .01, ***p < .001; *n* = 5 for each group).

Cigarette smoke exposure had no significant effect on GLT-1 protein expression in the PFC, but 30 mg/kg upregulated GLT-1 expression, as shown in Figure 6A. This pattern of effects was confirmed by one-way ANOVA revealing a significant main effect of Treatment [F (Association, 2014; Linker et al., 2019) = 10.07, *p* = .0016]. Tukey’s multiple comparisons showed a significant upregulation in GLT-1 expression in the NIC/ASA30 group compared to the NIC and NIC/ASA15 groups.

Cigarette smoke exposure decreased xCT protein expression in the PFC, and this decrease was reversed by treatment with 30 mg/kg ASA, as shown in Figure 6B. This pattern of effects was confirmed by one-way ANOVA revealing a significant effect of Treatment in the PFC [F (Association, 2014; Linker et al., 2019) = 13.64, *p* = .0005]. Tukey’s multiple comparisons showed a significant downregulation in xCT expression in the NIC and NIC/ASA15 groups compared to Control, NIC/ASA30, and ASA30 groups.

Finally, cigarette smoke exposure increased NF-κB protein expression, and this increase was reversed by treatment with 15 mg/kg or 30 mg/kg ASA as shown in Figure 6C. This pattern of effects was confirmed by one-way ANOVA that showed a significant main effect of Treatment [F (Association, 2014; Linker et al., 2019) = 34.95, *p* < .0001). Turkey’s multiple comparisons showed that there were significant increases in NF-κB protein expression in the NIC group compared with the Control, NIC/ASA15, NIC/ASA30, and ASA30 groups.

### 3.8 Effect of cigarette smoke exposure and ASA treatment on GLT-1, xCT, and NF-kβ protein expression in the NAc

**FIGURE 7:**
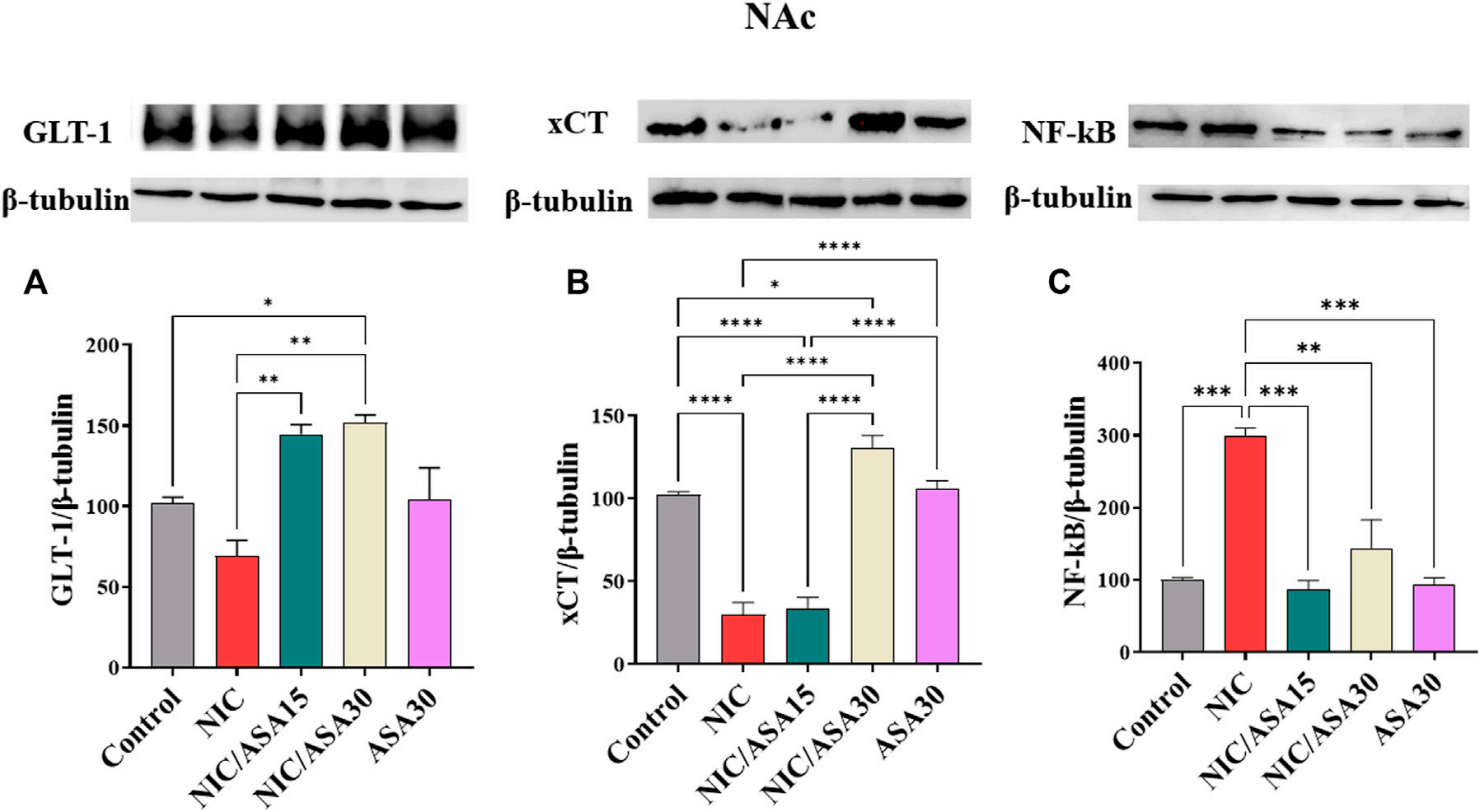
Glutamate transporters level in NAc brain regions after treatments (data expressed as mean ± SEM). **(A)** GLT-1; **(B)** xCT; **(C)** NFκB (*p < .05, **p < .01, ***p < .001; *n* = 5 for each group).

Cigarette smoke exposure did not significantly reduce levels of GLT-1 protein expression in the PFC. Treatment with 15 mg/kg ASA or 30 mg/kg ASA 45 min before cigarette exposure for 11 days up-regulated GLT-1 protein expression in the PFC, as shown in Figure 7A. This pattern of effects was confirmed by one-way ANOVA revealing a significant effect of Treatment [F (Association, 2014; Linker et al., 2019) = 10.40, *p* = .0014]. Tukey’s multiple comparisons revealed a significant increase in GLT-1 protein expression in the NIC/ASA15 and NIC/ASA30 groups compared to control and NIC groups. Related effects were found for xCT. Cigarette smoke exposure decreased xCT protein expression in the NAc, and this decrease was reversed by 30 mg/kg ASA but not 15 mg/kg ASA treatment, as shown in Figure 7B. This pattern of effects was confirmed by one-way ANOVA revealing a significant effect of Treatment [F (Association, 2014; Linker et al., 2019) = 58.62, *p* < .0001]. Tukey’s multiple comparisons showed that there was significant decrease in xCT protein expression in the NIC and NIC/ASA15 groups compared to the Control, NIC/ASA30 and ASA30 groups. Finally, cigarette smoke exposure increased NF-κB protein expression in the NAc, and this increase was reversed by treatment with 15 mg/kg and 30 mg/kg ASA, as shown in Figure 7C. This pattern of effects was confirmed by one-way ANOVA that showed a significant main effect of Treatment [F (Association, 2014; Linker et al., 2019) = 19.72, *p* < .0001]. Turkey’s multiple comparison showed that there were significant increases in NF-κB protein expression in the NIC group compared with the Control, NIC/ASA15, NIC/ASA30, and ASA30 groups.

## 4 Discussion

This study explored for the first time the effect of cigarette smoke exposure for 31 days as well as the effect of treatment for 11 days with ASA on nicotine withdrawal-induced anxiety, NF-κB expression, and glutamatergic transporter expression. The findings clearly indicate that cigarette smoke exposure affects glutamate transporter expression, alters NFκB expression and causes anxiety-like behavior.

Firstly, the current study showed that 2 h/day, 5 days/week, exposure to cigarette smoke for 31 days resulted in anxiety-like behavior in the OF and LD tests. This was indicated by decreased distance traveled and time spent in center of the OF, time spent in light chamber and the latency of the initial entry into the opposite compartment in the LD test. These results are consistent with previous animal studies showing that withdrawal from cigarette smoking or nicotine exposure has anxiogenic effects (de la Peña et al., 2016; Chellian et al., 2020) during the timeframe studied here (e.g., 24 h after the last nicotine/cigarette smoke exposure). Indeed, previous research has revealed that somatic symptoms steadily developed over time in male rats exposed to high-nicotine containing cigarette smoke, which shows that the amount of dependency grew with the duration of exposure (Chellian et al., 2020). Data showed that anxiety-like behavior increased in rats exposed to nicotine, as evidenced by decreased open arm duration, open arm entries, distance travelled, and time spent in center zone of an OF (Hammad et al., 2021). Increased anxiety-like behavior is also observed following adolescent nicotine exposure as assessed in an open field “conflict” test (Slawecki et al., 2003). Other investigations revealed anxiogenic responses to short-term nicotine abstinence in adult rats. For example, in adult rats, short-term abstinence (e.g., 24 h) following repeated nicotine injections produces anxiety-like responses in the elevated plus maze (Irvine et al., 2001; Iniguez et al., 2009).

Previous studies that showed ASA (15 mg/kg for 11 days) reduces chronic ethanol intake and relapse to binge-drinking (Israel et al., 2021), and attenuates reinstatement of oral nicotine intake (Quintanilla et al., 2020). Furthermore, several studies in rats and humans have shown that oral administration of ASA at doses lower than those utilized in the present study produce brain levels above those necessary for biological activity (Berk et al., 2013; Quintanilla et al., 2020; Israel et al., 2021). In addition to these findings, ASA has been shown to have beneficial effects for depression and mood disorders, schizophrenia, and Alzheimer’s disease (Berk et al., 2013), indicative of neural actions of ASA. In the present study, we found that 30 mg/kg ASA treatment, but not 15 mg/kg ASA treatment, attenuated cigarette smoke withdrawal-induced anxiety.

As much previous evidence suggests, withdrawal-induced anxiety involves both neuroinflammation and changes in glutamate homeostasis. The present study demonstrated that nicotine exposure *via* cigarette smoke downregulated GLT-1 gene and protein levels in the NAc, while tobacco smoke exposure decreased xCT gene and protein levels in the PFC and the NAc. Ample of evidence links anxiety-like behavior with changes in synaptic activity resulting from dysregulation of glutamatergic function (Ryu et al., 2021). A growing body of evidence demonstrates that glutamate neurotransmission plays a key role in anxiety disorder etiology as well (Garakani et al., 2006; Manhaes et al., 2008). Furthermore, glutamate neurotransmission has been related to anxiety in animal models (Bermudo-Soriano et al., 2012). Previous research has shown that GLT-1 downregulation is a consistent neuroadaptation resulting from chronic exposure to drugs of abuse across a range of drug classes (Garcia-Keller et al., 2016). Moreover, GLT-1 expression restoration is related to a reduction in cue-induced reinstatement of cocaine craving (de las Drogas, 2012), demonstrating that these changes have an important role in the maintenance of drug dependence. Additionally, several studies have shown that xCT can modulate extracellular glutamate concentrations (Savaskan and Eyüpoglu, 2010; Rao et al., 2015), and that it also plays a crucial role in the reinstatement of nicotine dependence and drug seeking behavior (Kalivas et al., 2009). These data suggest that xCT may be a potential therapeutic target for the treatment of nicotine addiction. Two earlier sub-chronic nicotine studies found no alterations in GLT-1 or xCT in the prefrontal cortex of rats given nicotine orally or through minipumps for 21 days (Redcay and Courchesne, 2008), or in mice exposed intermittently to nicotine-containing e-cigarette vapor (Alasmari et al., 2017). However, the present study demonstrated that a long period of 31 days of nicotine exposure *via* cigarette smoke downregulated GLT-1 in the NAc and xCT expression in the PFC and the NAc. In previous research, downregulation of GLT-1 and xCT protein expression was seen in the NAc of rats self-administering nicotine, and additionally restoration of GLT-1 expression was related to a reduction in nicotine self-administration (Knackstedt et al., 2009; Sari et al., 2016). After cue-induced nicotine reinstatement, GLT-1 protein expression was significantly reduced in the NAc (Namba et al., 2020).

Changes in glutamate homeostasis also seem to involve neuroinflammatory states. Smokers and COPD patients had higher levels of NF-κB expression and activation in their bronchial biopsies, which is associated with reduced airflow (Shao et al., 2019). The expression of NF-κB in lung tissue was also elevated in an animal model following exposure to cigarette smoke (D’souza and Markou, 2011). Earlier research from our laboratory has demonstrated enhanced NF-κB expression in mesocorticolimibic brain regions, including the PFC and the NAc in rats exposed to waterpipe tobacco smoke (Hammad et al., 2021). Consistent with those previous results, the present data suggests that after 31 days of cigarette smoke exposure an upregulation in NF-κB expression occurs in the PFC and the NAc. One limitation of the present work is that it did not confirm NF-κB activation and downstream effects. Future work should confirm whether this increased expression of NF-κB translates into activation of downstream mediators and other measures of neuroinflammation. This has been suggested to occur with other drugs of abuse such as ethanol (Nennig and Schank, 2017). Data such as this suggests that drugs of abuse alone produce neuroinflammatory states. Since the current study used tobacco smoke, it remains to be seen whether nicotine alone, or nicotine in other forms (such as vaping) produce the same effects as tobacco smoke. Cigarettes contain, or produce during the smoking process, at least 4,000 chemical components other than nicotine (the main psychoactive component of cigarettes) including, tar, carbon monoxide, arsenic, ammonia, acetone, toluene, methylamine, pesticide, polonium-210, acetaldehyde and methanol, many of which may contribute to neuroinflammatory effects (Hoffmann and Wynder, 1999). Additionally, other substances in cigarettes may play a role in the rewarding effects of smoking as well. Naturally occurring monoamine oxidase inhibitors and acetaldehyde in tobacco have been suggested to contribute to the reinforcing effects of nicotine and have been shown to enhance the reinforcing effects of nicotine (Levin et al., 2021). The contribution of these other substances to the reinforcing and adverse effects of smoking, which both may involve neuroinflammation will be important to understand for evaluating the relative risk of different nicotine products for substance abuse liability, as well as negative health outcomes.

Previous studies have suggested that cigarette smoke exposure can increase dopamine and glutamate secretion. The release of dopamine may be sufficient to trigger oxidative stress *via* spontaneous and enzymatic oxidation (Berríos-Cárcamo et al., 2020). Oxidative stress can inhibit GLT-1 and xCT expression through various mechanisms, including upregulation of TNF-α and IL1β, as well as activation of nuclear factor-E2-related factor 2 (Nrf2) (Kazama et al., 2020). Several studies have found that both brain oxidative stress and neuroinflammation downregulate GLT-1 and xCT (Quintanilla et al., 2020). Other studies suggest that prolonged nicotine exposure in rats is connected with elevated TNF-α levels in the PFC and NAc (Moylan et al., 2013). Recent rodent studies also found that chronic nicotine administration and nicotine withdrawal symptoms were linked to alterations in microglial morphology and microglial-mediated production of pro-inflammatory cytokines such as TNF-α and IL-1β in the striatum (Quintanilla et al., 2020).

A growing body of evidence suggests that glutamatergic neurotransmission plays an important role in the pathophysiology of anxiety disorders (Amiel and Mathew, 2007). Furthermore, alterations in glutamatergic neurotransmission have been related to the development of anxiety in various animal models. For instance, reduced GLT-1 expression was linked to anxiety-like behaviors during ethanol withdrawal in a rat model (Kang et al., 2018). By contrast, pharmacological treatments that normalize GLT-1 and xCT system activity appear to have beneficial effects on various drug dependence related outcomes. β-lactam antibiotics are powerful inducers of GLT-1 and xCT expression, dramatically increasing their mRNA and protein levels (Hammad and Sari, 2020), and these effects are associated with reduced drug intake, drug reinforcement, and withdrawal-induced effects (Hakami et al., 2016; Sari et al., 2016; Hammad and Sari, 2020; Hammad et al., 2021). Interestingly, a previous study has showed that ASA, taken at physiologically-relevant levels, reduces oxidative stress in the hippocampus of rats after chronic ethanol consumption (Israel et al., 2021). The treatment also lowered measures of brain oxidative stress, e.g., the ratio of oxidized glutathione to reduced glutathione in the hippocampus, and inhibited astrocyte and microglia activation. ASA stimulates the production of peroxisome proliferator-activated receptor-gamma (PPAR-γ), which is known to have powerful anti-inflammatory effects, as well as brain GLT-1 transcription, which increases GLT-1 protein levels and glutamate absorption (Yiqin et al., 2009). The data from the present study showed that the 15 mg/kg ASA dose was able to reverse some markers altered by cigarette smoke exposure (xCT and NF-kB mRNA levels in the PFC, GLT-1 and NF-kB mRNA and protein levels in NAc), but not others (xCT mRNA and protein levels in the NAc, and xCT protein levels in the PFC). Moreover, ASA did not affect the behavioral outcomes. This would seem to suggest that some of these changes, or perhaps all of the changes combined (e.g., both xCT and GLT-1) are necessary to reverse the behavioral outcomes of chronic cigarette smoke exposure. This is parallel to a previous study that showed that in order for ceftriaxone to attenuate relapse-like ethanol drinking, upregulation of both GLT-1 and xCT in NAc is required (Das et al., 2022). More studies are warranted to explore this effect, but it may point to the fundamental necessary involvement of both glutamate transporters. The present findings showed that ASA treatment (30 mg/kg) significantly increases mRNA and protein expression of GLT-1 and xCT in the NAc and PFC after nicotine exposure *via* cigarette smoke for 31 days, and it was under these conditions that alterations in behavior were observed.

Overall, these findings suggest that treatments targeting neuroinflammatory processes, such as ASA, may be useful treatments for nicotine-induced withdrawal effects, and potentially for nicotine dependence. These effects involve a link between neuroinflammatory processes and glutamate homeostasis.

## Data Availability

The original contributions presented in the study are included in the article/Supplementary Materials, further inquiries can be directed to the corresponding author.

## Abbreviations

ASA, acetylsalicylic acid; GLT-1, glutamate transporter; LD, light and dark box; LPS, lipopolysaccharide; NAc, nucleus accumbens; NF-κB, nuclear transcription factor-κB; NIC, nicotine; OF, open field; PFC, prefrontal cortex; TNF-α, tumor necrosis factor alpha; VTA, ventral tegmental area; xCT, glutamate–cystine antiporter.
