# Supplementary figures and images for: Acetylsalicylic acid reduces cigarette smoke withdrawal-induced anxiety in rats via modulating the expression of NFĸB, GLT-1, and xCT
Source: Front Pharmacol. 2023 Jan 9;13:1047236. doi: 10.3389/fphar.2022.1047236 (PMC9868824; doi:10.3389/fphar.2022.1047236)

## Slide 1
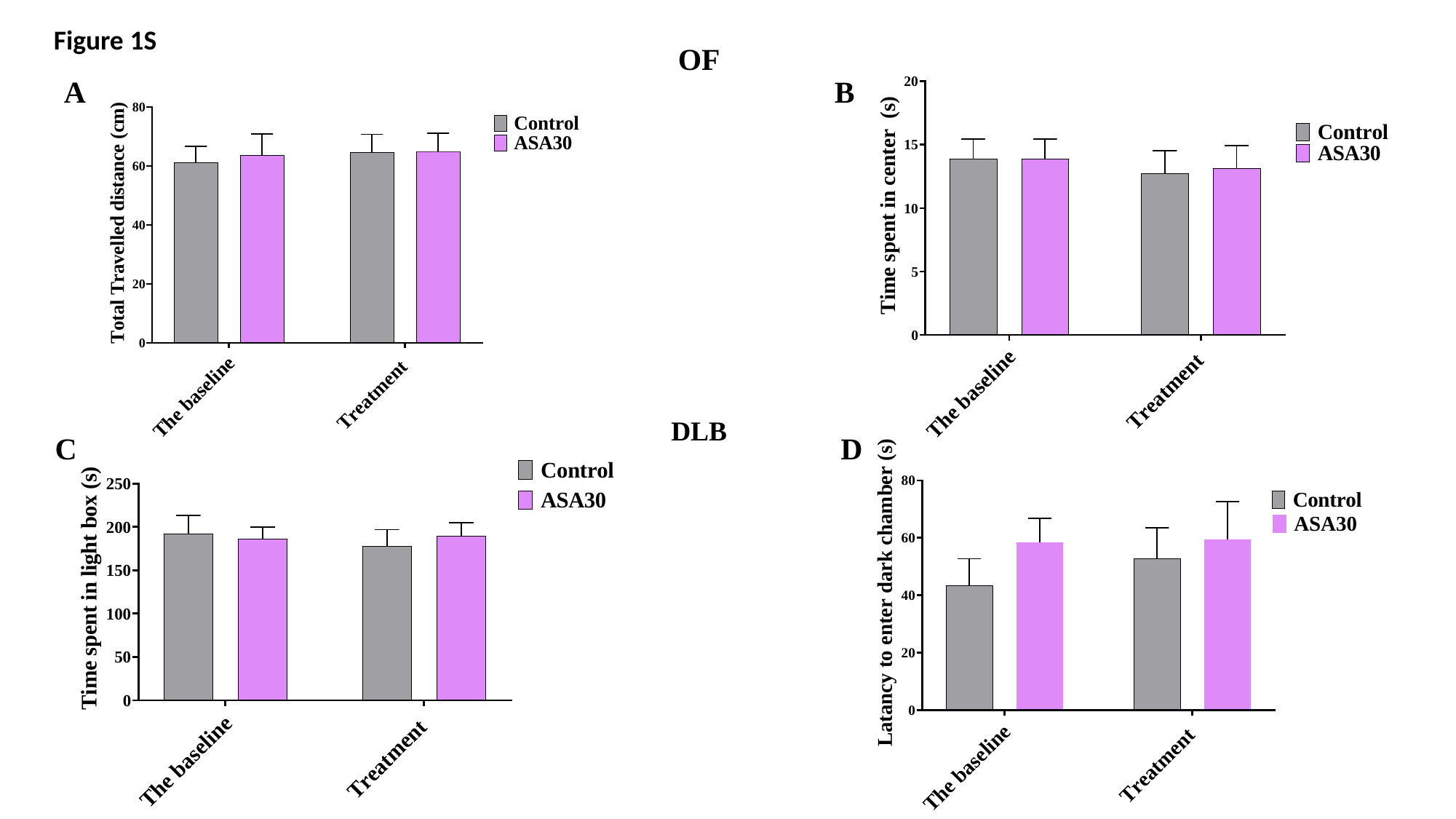

Figure 1S
OF
A
B
DLB
C
D

Supplement: Supplementary file 1 [file Presentation1.PPTX]
